# Misdiagnosed Uterine Rupture of an Advanced Cornual Pregnancy

**DOI:** 10.1155/2012/289103

**Published:** 2012-04-03

**Authors:** Christian Linus Hastrup Sant, Poul Erik Andersen

**Affiliations:** Department of Radiology, Odense University Hospital, Sdr. Boulevard 29, 5000 Odense C, Denmark

## Abstract

Cornual pregnancy is a diagnostic and therapeutic challenge with potential severe consequences if uterine rupture occurs with following massive intraabdominal bleeding. We report a case of a misdiagnosed ruptured cornual pregnancy occurring at 21 weeks of gestation. Ultrasound examination and computer tomography revealed no sign of abnormal pregnancy. The correct diagnosis was first made at emergency laparotomy. Uterine rupture should be considered in pregnant women presenting with abdominal pain and haemodynamic instability.

## 1. Introduction

Gestation in one horn of a bicornuate uterus is a rare form of pregnancy that poses a diagnostic and therapeutic challenge. We present a misdiagnosed ruptured pregnancy in a bicornuate uterus.

## 2. Case Story

A 30-year-old woman, gravida 2, para 0, presented at the Emergency Department with syncope and diffuse abdominal pain at gestation week 21 + 5. She did not have other abdominal or urinary symptoms and no vaginal bleeding.

She had visited the emergency department twice before within the previous week with similar symptoms and both times she was discharged within 24 h after physical examination and transvaginal ultrasound (TVUS).

Two years before, she had undergone examination for primary infertility, which showed a normal-sized anteverted uterus with no mentioning of uterine anomalies. She was diagnosed and treated for polycystic ovarian syndrome. She had a past gynecological history with termination of a pregnancy in her teens and had been treated for chlamydia.

The actual pregnancy was conceived spontaneously and she did not take any medication. On physical examination, she was in haemodynamic shock with blood pressure 59/30 and heart rate 140. Her abdomen was adipose with diffuse tenderness with maximum tenderness in the upper right quadrant but no guarding or rebound tenderness was noted. There was no vaginal bleeding.

Blood pressure was normalized after supportive treatment with fluid and blood, and analgesics were initiated but the patient remained tachycard.

Subsequent TVUS examination showed intrauterine pregnancy with foetus measurements consistent with gestational age. Transabdominal ultrasound examination revealed a 7 mm stone in the cystic duct and a thick-walled gallbladder but nondilated bile ducts.

Laboratory tests indicated anemia (hemoglobin level 5,8 mmol/L, erythrocyte vol.fr. 0,21) and possible infection (white blood cell count 22,5 10E9/l and C-reactive protein 35 mg/L). Liver blood tests were normal.

A provisional diagnosis of cholecystitis was made and treatment with antibiotics was instituted.

**Figure 1:**
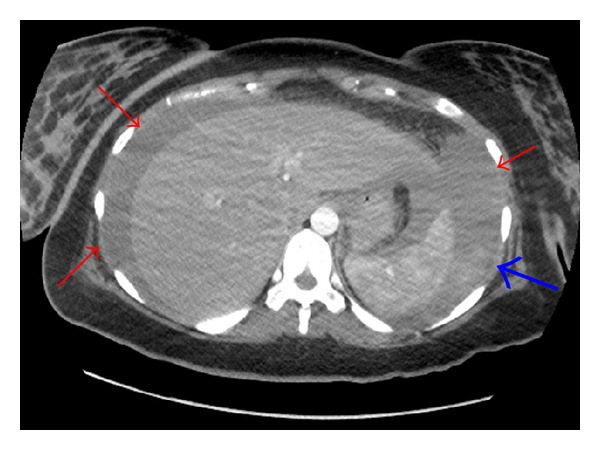
Massive intraperitoneal bleeding located around the liver and spleen (arrows) and suspected spleen blushing (thick arrow).

**Figure 2:**
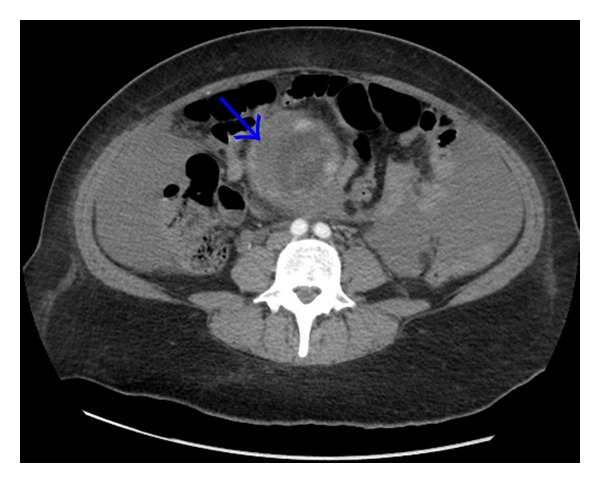
Retrospectively, bleeding could be seen from the right uterine corner (thick arrow).

**Figure 3:**
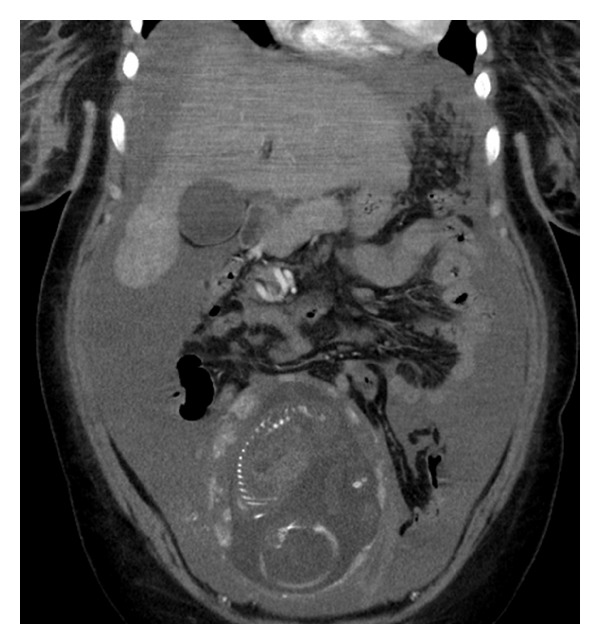
Massive hemoperitoneum and pregnant uterus. The pregnancy looks like a normal intrauterine pregnancy.

After 24 h, she still had diffuse abdominal pain and was tachycard. Laboratory tests demonstrated dropping hemoglobin level (4,5 mmol/L) despite resuscitation. Intraabdominal bleeding was suspected and esophago-gastro-duodenoscopy was performed with no sign of bleeding. Transabdominal ultrasound was repeated and revealed free pelvic fluid. At diagnostic ascites puncture dark bloody fluid was extracted. Abdominal computed tomography (CT) raised suspicion of splenic rupture and the patient was transferred to university hospital with the intention to embolize the spleen (Figures 1, 2, and 3). However, coeliacography showed no bleeding around the spleen.

On suspicion of ongoing abdominal bleeding, an emergency diagnostic laparotomy was performed and approximately 5 L of intraabdominal bloody fluid was evacuated, and a nonmural rupture of a right cornual pregnancy with placenta accreta in a bicornuate uterus was recognized. Normal conditions around the spleen and intestines were found. Peroperative ultrasound scanning showed a living fetus in the right uterine corner and right cornual resection and salpingectomy was performed. There was connection between both horns and the cervical canal. Bleeding during the operation amounted to more than 7 L and the patient was substituted with 11 units of packed red blood cells and 5.5 L of thin fluids. However, the foetus died during the operation.

The patient had an uneventful recovery and was discharged after 6 days. Hysteroscopy has later shown a normal-sized uterine cavity. As Müllerian abnormalities are associated with congenital kidney malformations, a CT urography was performed as well and demonstrated normal kidneys. 

## 3. Discussion

The prevalence of congenital uterine malformations is about 6,7% in the female population and higher in women with reproductive problems [1].

Bicornuate uterus is an abnormality with a partial nonfusion of the Müllerian duct resulting in a central myometrium that can extend as far down as the internal cervix opening. This malformation makes up approximately 3% of the uterine malformations [1].

The malformation in itself is asymptomatic but is associated with an increased rate of reproductive problems including repeated late abortions or miscarriages [2, 3].

The anomaly is often overlooked in routine gynecological examinations. Sonohysterography has been suggested as a screening tool to identify congenital malformations in infertile patients, as 2D ultrasound examination, diagnostic hysteroscopy, or hysterosalpingography all have a low sensitivity [1, 4].

The gold standard is combined laparoscopy and hysteroscopy to differentiate the malformation from other congenital uterine abnormalities. Magnetic resonance imaging (MRI) and 3D ultrasound are considered comparable to laparoscopy/hysteroscopy as less invasive alternatives but still with a high sensitivity [4].

The patient history of subfertility should have raised suspicion of a possible uterine anomaly in this case, but the patient also had other possible explanations for reduced fertility (e.g., adipose, polycystic ovarian syndrome and previous chlamydia infection).

Ectopic pregnancy is defined as pregnancy outside the endometrium of the uterus. 1.5–2% of all pregnancies are ectopic and ectopic implantation is a leading cause of pregnancy-related deaths [5].

Cornual pregnancy is pregnancy implanted in the upper lateral portion of a bicornuate or septate uterus [6].

In literature cornual pregnancy is used interchangeably with angular and interstitial pregnancies [7–10], although the aforementioned conditions are very different entities with different clinical presentations and treatment [11].

Angular pregnancy is an intrauterine pregnancy with a gestation implanted lateral in the uterine cavity medial to the uterotubal angle and the round ligament [12]. Though it is actually an intrauterine pregnancy, angular pregnancy is a potentially dangerous condition associated with uterine rupture, often in the second trimester [12]. The clinical course of an angular pregnancy is very variable with some full term pregnancies and many spontaneous miscarriages [11–13].

Interstitial pregnancy is an ectopic pregnancy implanted intramurally in the proximal part of the fallopian tube laterally to the round ligament. This ectopic pregnancy typically presents and ruptures before gestation week 12, much like the more common tubal ectopic pregnancy. It is associated with higher mortality than tubal pregnancies though [11].

An early diagnosis of ectopic pregnancy is important as a delay in the correct diagnosis is known to increase the risk of maternal morbidity and mortality [14].

An early ectopic pregnancy can be diagnosed accurately with repeated serum human chorionic gonadotropin tests combined with TVUS with sensitivity >90% [15] although ultrasound is dependent on the operator and the gestational age [16].

Correct diagnosis of an advanced extrauterine pregnancy warrants a high index of suspicion because symptoms are often nonspecific and diagnostic imaging inconclusive. Ultrasound imaging is considered the most important diagnostic tool and MRI can be made as an additional examination but even then diagnostic accuracy is low [17]. Best results obtained were reported in a case series from 2007 were 6 of 10 patients where discovered preoperatively [18].

A cornual pregnancy is also difficult to diagnose preoperatively with low ultrasonographic sensitivity [11]. It is easily confused with tubal ectopic pregnancy or a normal intrauterine pregnancy. Recent case series have shown preoperative diagnosis of cornual pregnancies below 70% [19, 20].

It is even more difficult to differentiate between angular and interstitial pregnancy.

Sonographically, interstitial pregnancies are recognizable if an empty uterine cavity is found together with an eccentric placed gestational sac and a thin myometric mantle. By including the “interstitial line sign” as a parameter sensitivity can be increased [11].

It is anecdotally reported that 3D ultrasound and MRI can give more accurate information about the exact position of the gestational sac and thus help to differentiate between angular and interstitial pregnancies [21, 22].

In this case, no conclusion could be made if the cornual pregnancy was an eccentric intrauterine (angular) pregnancy or it was an ectopic intramural (interstitial) pregnancy.

The late presentation in gestation week 21 speaks for an eccentric intrauterine pregnancy as interstitial pregnancy typically presents earlier [11, 20].

TVUS, transabdominal ultrasound, and abdominal CT were performed in this case of advanced cornual pregnancy without rising suspicion of an abnormal pregnancy. Here ultrasonography was actually misleading as the pregnancy was interpreted as a normal intrauterine pregnancy and furthermore a gallstone was revealed in the cystic duct thus delaying the correct diagnosis.

The initial diagnosis of cholecystitis was revised when the patient developed symptoms of hypovolemic shock and intraabdominal bleeding was recognized ultrasonographically. Unfortunately the correct diagnosis was again delayed as splenic rupture was suspected on subsequent abdominal CT.

Abdominal CT is usually avoided in pregnancy except in emergency life threatening cases because of the potentially increased risk of cancer when exposing the fetus to ionizing radiation especially in the first trimester [23–26]. Spontaneous splenic rupture in a pregnant woman is a rare condition but has been described in the literature [27, 28]. Other causes of acute abdominal pain such as pancreatitis, cholecystitis, and appendicitis can simulate uterine rupture, which, however, should be considered in a pregnant woman presenting with abdominal pain and without vaginal bleeding.

MRI could have been used here as an alternative to CT but the patient was haemodynamically unstable and CT was chosen because of availability and speed.

Management of a cornual pregnancy depends on many factors including gestational location and age, haemodynamic status, presence of uterine rupture, and local factors such as the surgeons' expertise and preference and the patients' wishes of retaining fertility [11, 19, 20].

An advanced ruptured cornual pregnancy as described in this case is always a medical emergency and should be treated with laparoscopic or laparotomic surgery.

In this case, the decision of laparotomy was chosen because the patient was unresolved and haemodynamically unstable.

## 4. Conclusion

Laparotomy or laparoscopy is essential in the haemodynamically unstable advanced pregnant woman with abdominal pain to treat hemorrhage and avoid diagnostic dilemmas.
